# Psychotropic medications in older people in residential care facilities and associations with quality of life: a cross-sectional study

**DOI:** 10.1186/s12877-018-0752-0

**Published:** 2018-02-26

**Authors:** Stephanie L. Harrison, Clare Bradley, Rachel Milte, Enwu Liu, Lisa Kouladjian O’Donnell, Sarah N. Hilmer, Maria Crotty

**Affiliations:** 1 Department of Rehabilitation, Aged and Extended Care, Faculty of Medicine, Nursing and Health Sciences, Flinders University, Level 4, Rehabilitation Building, Flinders Medical Centre, Flinders Drive, Bedford park, Adelaide, SA 5042 Australia; 2 NHMRC Cognitive Decline Partnership Centre, The University of Sydney, Sydney, NSW Australia; 3 Infection & Immunity – Aboriginal Health, SAHMRI, PO Box 11060, Adelaide, SA 5001 Australia; 4 Institute for Choice, University of South Australia, GPO Box 2471, Adelaide, SA 5001 Australia; 5 Mary MacKillop Institute for Health Research, Australian Catholic University, Melbourne, VIC 3000 Australia; 6 Kolling Institute of Medical Research, University of Sydney and Royal North Shore Hospital, St Leonards, NSW 2065 Australia

**Keywords:** Psychotropic medications, Antipsychotics, Antidepressants, Quality of life, Residential care, Dementia

## Abstract

**Background:**

Psychotropic medications have been associated with many adverse outcomes in older people living in residential care. Home-like models of residential care may be preferable to traditional models of care and we hypothesized that this model may impact on the prevalence of psychotropic medications. The objectives were to: 1) examine associations between psychotropic medications and quality of life in older adults living in residential care facilities with a high prevalence of cognitive impairment and dementia and 2) determine if there was a difference in prevalence of psychotropic medications in facilities which provide a small group home-like model of residential care compared to a ‘standard model’ of care.

**Methods:**

Participants included 541 residents from 17 residential aged care facilities in the Investigating Services Provided in the Residential Environment for Dementia (INSPIRED) study. Cross-sectional analyses were completed to examine the above objectives. Quality of life was measured with the dementia quality of life questionnaire (DEMQOL) and the EQ-5D-5L completed by the resident or a proxy.

**Results:**

Overall, 70.8% (*n* = 380) of the population had been prescribed/dispensed at least one psychotropic medication in the 100 days prior to recruitment. An increased number of psychotropic medications was associated with lower quality of life according to DEMQOL-Proxy-Utility scores (β (SE): − 0.012 (0.006), *p* = 0.04) and EQ-5D-5L scores (− 0.024 (0.011), *p* = 0.03) after adjustment for resident-level and facility-level characteristics. Analysis of the individual classes of psychotropic medications showed antipsychotics were associated with lower DEMQOL-Proxy-Utility scores (− 0.030 (0.014), *p* = 0.03) and benzodiazepines were associated with lower EQ-5D-5L scores (− 0.059 (0.024), *p* = 0.01). Participants residing in facilities which had a home-like model of residential care were less likely to be prescribed psychotropic medications (OR (95% CI): 0.24 (0.12, 0.46), *p* < 0.001).

**Conclusions:**

An increased number of psychotropic medications were associated with lower quality of life scores. These medications have many associated adverse effects and the use of these medications should be re-examined when investigating approaches to improve quality of life for older people in residential care. Home-like models of residential care may help to reduce the need for psychotropic medications, but further research is needed to validate these findings.

**Electronic supplementary material:**

The online version of this article (10.1186/s12877-018-0752-0) contains supplementary material, which is available to authorized users.

## Background

In Australia in 2017 365,000 people were estimated to be living with dementia and almost half of these people were living in cared accommodation such as residential aged care facilities [1]. People who live in residential aged care facilities are more likely to experience a reduced quality of life compared to those living in the community [2]. Although quality of life does not always decrease as dementia progresses, degeneration of cognitive function and mood often reduces quality of life amongst people living with dementia [3].

Psychotropic medications include antipsychotics, antidepressants and benzodiazepines and these are commonly prescribed to older people in residential settings, particularly those with dementia, to manage behavioural and psychological symptoms of dementia (BPSD), despite limited evidence of efficacy in this setting and their known potential serious adverse effects [4, 5]. Advancing age and polypharmacy both increase the risk of these effects [6]. Evidence has suggested these medications may be over prescribed in residential aged care facilities in many countries, including Australia [7]. Potentially inappropriate use of psychotropic medications is a particular concern for people living in residential aged care as psychotropic medications have been associated with increased risk of falls, hospitalization, stroke and mortality in this population [7].

The physical environment of residential aged care facilities may affect quality of life for residents [8]. Research has suggested the development of a ‘home-like’ model of care which creates a person-centred environment and endeavours to facilitate independence for the residents may improve outcomes for residents, their family members and staff [9, 10]. These home-like models aim to provide a familiar environment for the resident to ease the transition from their family home to residential care. Establishing the home-like environment may involve different methods such as providing smaller living units with only 10 or 15 residents grouped together, increasing access to outdoor areas, encouragement and support for residents participating in domestic tasks (such as meal preparation), allocation of staff to the living unit routinely, home-like furnishings and institutional ‘presences’ such as medication carts, laundry trolleys removed or minimised. However, further research is needed to gain a clear understanding of the effect of home-like models of care on health-related outcomes of residents [11]. In this study we examined if there is a difference in the prevalence of psychotropic medications in residential facilities with a home-like model of care compared to a standard model of care as we hypothesized that these person-centred environments may reduce the need for these medications.

Previous studies have suggested an association between potentially inappropriate medications and lower quality of life in older people in residential care [12, 13]. However, there is limited evidence of the effect of the broad range of psychotropic medications on quality of life in people residing in aged care facilities with high levels of cognitive impairment and dementia. The objectives were to examine the association between psychotropic medications and quality of life in the Investigating Services Provided in the Residential Environment for Dementia (INSPIRED) study and also to examine if there was a difference between psychotropic medication use in a home-like model of residential care compared to a more standard model of care.

## Methods

### Study participants

The INSPIRED study is a cross-sectional study of people living in residential aged care facilities in Australia and included participants with cognitive impairment and dementia. The study received ethical approval from the Flinders Social and Behavioural Research Ethics Committee (Approval numbers 6732 and 6753). Briefly, the study included 541 long-term permanent residents (resided at the facility for 12 months or more) from 17 different facilities in four different states in Australia and participants either self-consented to be part of the study or a proxy (a close family member) provided consent on their behalf when the participant had moderate to severe cognitive impairment (PAS-Cog score ≥ 10). Further details of the INSPIRED study have been reported elsewhere [14].

### Measurement of medication use

Retrospective pharmacy dispensing records from the 100 days prior to the study start date for their facility were used to determine medication use. Recent prescription of a psychotropic medication referred to dispensing of the medication in the 100 day period. However, 3.5% (*n* = 19) of the participants had missing pharmacy records and therefore reviews of their medication charts were undertaken instead. No medication data were available for four of the participants, so the total sample size was 537 (99.3%). Psychotropic medications were defined based on a list of psychotropic medications from the Australian Medicines Handbook [15]. The medications were further classified in to three groups: antipsychotics, antidepressants and benzodiazepines.

### Resident-level characteristics

Data collected from the participants included Psychogeriatric Assessment Scale-Cognitive Impairment Scale (PAS-Cog) scores, modified Barthel Index scores and Neuropsychiatric Inventory (NPI) scores. To assess quality of life, a dementia specific measure of health-related quality of life was applied: the DEMQOL (self-completed by the participant) and the DEMQOL-Proxy (completed by a proxy for the participant). The DEMQOL is the only dementia specific quality of life instrument currently to have a set of utility weights developed, allowing it to be applied in cost-utility studies for evaluating the economic effectiveness of health and care interventions [16, 17]. Utility scores for the DEMQOL and DEMQOL-Proxy were then generated by applying preference-weights generated from the valuation study [17]. A measure of generic health-related quality of life (the EQ-5D-5L) was also undertaken (self-completion or proxy) [18]. Utility scores for the EQ-5D-5L were generated using preference-weights from a previous study [19]. The EQ-5D is the most commonly used quality of life scale in health research and as a generic preference based measure of quality of life it can be validly applied across participant groups and intervention types. The EQ-5D has been extensively studied and has been shown to have excellent reliability and validity, while the new 5 level version (as used in the current study) has been shown to reduce ceiling effects seen in people with minor health related quality of life impairments [18, 20].

### Facility-level characteristics

Data collected regarding the residential aged care facility included information from a standardised questionnaire (previously validated in a residential aged care population [21]). The questionnaire covers general characteristics, the living and care concept, living-unit staffing and the physical environment.

### A home-like model of residential care in the INSPIRED study

The INSPIRED study includes some facilities which have adopted a small group home-like environment of residential care. In the INSPIRED study, these home-like facilities were identified if the living units had five or more of six characteristics including smaller size (up to 15 residents), independent access to outdoors, care staff allocated to living units, meals cooked in the living units, self-service option for meals and residents given the option to assist with meal preparation [22, 23]. Further details of how these criteria were developed have been described elsewhere [24].

### Statistical analysis

The data had a two-level hierarchal structure as the residents were clustered in residential aged care facilities. Multi-level linear models were used to test for associations between psychotropic medication use and continuous test scores (quality of life measures, PAS-Cog scores and NPI scores). Logistic regression models were used to examine associations with a home-like model of care. Models were adjusted for potential confounding factors: facility-level characteristics (model of care: home-like or standard, location, number of direct care hours per resident, facility costs per resident and size of facility) and resident-level characteristics (age, sex, marital status, PAS-Cog scores, number of co-morbid conditions, NPI scores, social ties and activities of daily living). *P* < 0.05 was regarded as statistically significant. All analyses were completed using Stata v.14.0 (Stata Corp LP, College Station, TX, USA).

## Results

### Characteristics of the participants

The mean (SD) age of the participants was 85.5 (8.5) years old and 74.5% (*n* = 403) were female (Table 1). These figures are comparable with characteristics of those residing in permanent aged care in Australia, where the average age is 84.5 years old and 69% are female [25]. The median (IQR) PAS-Cog score of the participants was 15 (6–21) and a dementia diagnosis was recorded for 64.3% (*n* = 348) of the participants. This is higher than for the permanent aged care population of Australia, where 52% of the population have a diagnosis of dementia. Of the participants involved, 83% (*n* = 448) had some reported level of cognitive impairment, ranging from mild to severe.

**Table 1:** Characteristics of the participants of the INSPIRED Study

Characteristic	All participants (*n* = 541)	Participants residing in a home-like model of care (*n* = 120)	Participants residing in a standard model of care (*n* = 421)	*P* value
Participant characteristics				
Age, mean (SD)	85.5 (8.5)	83.3 (9.0)	86.1 (8.3)	0.001
Female, n (%)	403 (74.5)	90 (75.0)	313 (74.4)	0.89
Married, n (%)	137 (25.4)	36 (30.0)	101 (24.1)	0.19
Barthel Index, median (IQR)	35 (9–71)	34 (5–66)	36 (11–72)	0.18
NPI, median (IQR)	7 (3–12)	10 (6–15)	6 (3–12)	< 0.001
PAS-Cog, median (IQR)	15 (6–21)	21 (15–21)	12 (4–21)	< 0.001
Number of co-morbid conditions, mean (SD)	3.7 (1.4)	3.2 (1.4)	3.8 (1.4)	< 0.001

*Abbreviations*: *IQR* Inter-quartile range, *P* values are from chi-squared or Mann-Whitney tests

### Psychotropic medication use amongst the participants

There were 380 participants (70.8%) who had been prescribed/dispensed one or more psychotropic medications in the 100 days prior to the study start date. The number of psychotropic medications a person was exposed to ranged from 0 to 5 psychotropic medications. The median (IQR) total number of different medications (regular and as needed, prescription and over the counter) that participants were prescribed was 10 (7–13), of which 10.0% (0%–18.8%) were psychotropic medications. For the individual classes of psychotropic medications, the following prevalence rates were observed: antidepressant 49.9% (*n* = 268), antipsychotic 24.8% (*n* = 133) and benzodiazepine 30.5% (*n* = 164). A list of the different psychotropic medications prescribed to the participants is shown in the Additional file 1: Table S1.

### Psychotropic medications and a home-like model of residential care

Of the 17 facilities which participated, 4 facilities (23.5%) were classified as a home-like model of residential care (22.2% (*n* = 120) residents living in a home-like model and 77.8% (*n* = 421) residents living in a standard model). Of those who resided in a home-like model of care, 98.3% (*n* = 118) had a diagnosis of dementia. Participants residing in a home-like model of care were younger, had higher NPI scores, had higher (worse) PAS-Cog scores and had fewer co-morbid conditions than those living in a standard model of residential care (*p* < 0.05) (Table 1).

The proportion of people taking a psychotropic medication in a home-like model was 61.7% and residents were significantly less likely to be taking a psychotropic medication in a home-like model compared to those in a ‘standard model’ (OR (95% CI) 0.24 (0.12, 0.46), *p* < 0.001), after adjustments (Table 2). These participants were also less likely to be taking antidepressants (0.50 (0.28, 0.88), *p* = 0.02) and benzodiazepines (0.11 (0.05, 0.24), *p* < 0.001).

**Table 2:** Psychotropic medications in a home-like model of residential care compared with standard models of care

	Home-like model, OR (95% CI), *P* value
Psychotropic medication	0.24 (0.12, 0.46), < 0.001
Antipsychotic medication	0.67 (0.34, 1.33), 0.25
Antidepressant medication	0.50 (0.28, 0.88), 0.02
Benzodiazepine medication	0.11 (0.05, 0.24), < 0.001

The reference for prescribed one or more psychotropic medications is being prescribed no psychotropic medications. The models are adjusted for facility-level confounding factors: location, number of direct care hours, facility cost and size of facility and resident-level characteristics: age, sex, marital status, PAS-Cog scores, number of co-morbid conditions, NPI scores, social ties and Barthel Index

### Psychotropic medications and quality of life

The use of psychotropic medications was not associated with quality of life according to any of the quality of life measures, but an increasing number of psychotropic prescriptions was associated with significantly lower EQ-5D-5L scores (β (SE) after adjustment for potential confounding factors: − 0.024 (0.011), *p* = 0.03) and significantly lower DEMQOL-Proxy-Utility scores (− 0.012 (0.006), *p* = 0.04) (Table 3).

**Table 3:** Associations between psychotropic medication use and quality of life, cognitive function and behaviour

	Psychotropic medication, β (SE), *P* value	Increasing number of psychotropic medications β (SE), *P* value	Antipsychotic medication, β (SE), *P* value	Antidepressant medication, β (SE), *P* value	Benzodiazepine medication, β (SE), *P* value
Quality of life					
DEMQOL proxy scores (*n* = 535)	−0.020 (0.013), 0.10	−0.012 (0.006), 0.04	− 0.030 (0.014), 0.03	− 0.018 (0.011), 0.11	− 0.008 (0.013), 0.53
DEMQOL scores of the residents (n = 228)	− 0.024 (0.017), 0.16	− 0.009 (0.009), 0.32	−0.040 (0.025), 0.11	− 0.009 (0.017), 0.61	−0.016 (0.018), 0.37
EQ-5D-5L scores (resident or proxy) (*n* = 531)	−0.018 (0.023), 0.44	−0.024 (0.011), 0.03	− 0.031 (0.026), 0.22	0.003 (0.021), 0.88	− 0.059 (0.024), 0.01
Behaviour					
NPI (*n* = 534)	1.571 (0.506), 0.002	1.072 (0.229), < 0.001	2.525 (0.541), < 0.001	0.495 (0.463), 0.29	1.443 (0.514), 0.005
Cognitive function					
PASCog (n = 537)	0.979 (0.577), 0.09	0.752 (0.261), 0.004	2.650 (0.584), < 0.001	−0.401 (0.499), 0.42	−0.136 (0.560), 0.81

The reference for prescribed one or more psychotropic medications is being prescribed no psychotropic medications. The number of psychotropic medications was analysed as a continuous score. The models are adjusted for facility-level confounding factors: model of care, location, number of direct care hours, facility cost and size of facility and resident-level characteristics: age, sex, marital status, PAS-Cog scores (except where the outcome is PASCog scores), number of co-morbid conditions, NPI scores (except where the outcome is NPI scores), social ties and Barthel Index

*Abbreviations*: *DEMQOL* Dementia quality of life questionnaire, *EQ-5D-5L* EuroQol five dimensions questionnaire, *NPI* neuropsychiatric inventory, *PASCog* Psychogeriatric Assessment Scales – Cognition

When examining the DEMQOL self-report scores (*n* = 228), psychotropic medications (− 0.024 (0.017), *p* = 0.16) and an increasing number of psychotropic medications (− 0.009 (0.009), *p* = 0.32) were not associated with lower quality of life after adjusting for confounding factors.

### Different classes of psychotropic medications and quality of life

When looking at the psychotropic medications by different classes of drugs, antipsychotic medications and benzodiazepines were associated with some measures of reduced quality of life. Antipsychotic medications were associated with lower DEMQOL-Proxy-Utility scores after adjustment for confounding factors (− 0.030 (0.014), *p* = 0.03). Benzodiazepines were associated with reduced quality of life according to the EQ-5D-5L (− 0.059 (0.024), *p* = 0.01).

## Discussion

In this cross-sectional study of older adults with high levels of cognitive impairment and dementia, living in Australian residential care facilities, higher exposure to psychotropic medications was associated with lower quality of life. Antipsychotics and benzodiazepines were both independently associated with lower quality of life. In facilities which had adopted a small group home-like model of residential care, there was a lower prevalence of psychotropic medications.

In this study, 24.8% of the total population and 35.9% of the population with a diagnosis of dementia had been prescribed an antipsychotic recently. This is in line with rates of antipsychotic medications reported previously in Australian residential aged care facilities which have shown prevalence rates of antipsychotics of 30.2% [26] for all residents, and 28.2% [27] to 44% [28] for residents with dementia. The rate for benzodiazepines (30.5%) was similar for those with or without a diagnosis of dementia and this is higher than in previous studies of residential aged care facilities in Australia: 11.4% for all residents [26] and 18.8% in residents with dementia [27]. Residents living in facilities which had adopted a home-like model of residential care were less likely to be prescribed benzodiazepines and antidepressants. Further research should investigate how these facilities are managing to limit the use of benzodiazepines and antidepressants, despite the high proportion of people residing in these facilities with dementia.

Psychotropic medications can have many adverse effects including cardiovascular complications and adverse mental state changes [15]. Recommendations have been made to avoid the use of certain medications in older people and people with dementia, including antipsychotics and benzodiazepines [29]. However, people with dementia are very likely to experience BPSD at some point and this can be a significant challenge for staff in residential care [30]. Recent clinical practice guidelines for people with dementia suggest non-pharmacological interventions should be used as a first line treatment for changed behaviours, such as person-centred care approaches, individual care plans and specific therapies [31]. Despite such recommendations, previous studies have suggested there may be inappropriate prescribing of psychotropic medications [32]. As demonstrated in this study, psychotropic medications may be associated with lower quality of life. People who were prescribed a psychotropic medication also had higher levels of neuropsychiatric problems and cognitive impairment. Those taking psychotropic medications may have lower quality of life due to their behavioural symptoms and the results here may highlight that the current pharmacological treatments are not managing their behavioural symptoms effectively.

Facilities which had adopted a home-like model of residential care were shown to have a lower prevalence of psychotropic medications even though the residents of these facilities were more likely to have dementia, cognitive impairment and higher NPI scores. However, individuals residing in a home-like model were also more likely to be younger and have fewer co-morbid conditions. Nevertheless, these characteristics were adjusted for and therefore these are unlikely to have influenced the results of this study. Home-like small group models of care in different countries, such as the Green House model in the US, may improve certain outcomes for residents such as quality of life, behavioural symptoms and well-being [9, 33]. In Europe, research has suggested these home-like models of residential care for people with dementia may improve physical functioning of the residents [11]. However, the effectiveness of small group home-like models on different health-related outcomes for residents needs to be further explored [11]. The person-centred home-like environment provided in a home-like model compared to a standard model of care may be impacting on their management of BPSD, which may be reducing the use of psychotropic medications, but this would need to be further explored. Specific aspects of the home-like model which may be reducing the need for psychotropic medications could be the home-like environment is more familiar, being similar to their family home, that staff assigned to the living unit may be better equipped to respond to the challenges seen with people with BPSD without the need for psychotropic medications, or the home-like model is a more stimulating environment and is better for the resident (e.g., increased outdoor access and option to help with meal preparation). The exact reasons for why the home-like model may be associated with a lower prevalence of psychotropic medications would be a useful question for further research in order to improve the evidence-base to guide management approaches in residential care.

The prescriptions of psychotropic medications to older residents in residential aged care facilities need frequent review to see if the potential harms, which our study suggests may include reduced quality of life, are outweighing the benefits of the medications and if alternative non-pharmacological approaches could be used. Functional-analysis based interventions have been shown to have a significant effect on reducing global BPSD with moderate quality evidence and without the adverse events seen with psychotropic medications [34].

### Strengths

Although cross-sectional in nature, this is a relatively large cohort study in this context, and by including those who could not consent for themselves we were able to include people with moderate to severe dementia. Data were collected not only for the residents, but also for the facilities in which they resided and there was minimal missing data for the participants. This allowed for adjustment of a wide-range of potential confounding factors relating to both the resident and their residential facility. Furthermore, to assess quality of life we used two different validated scales, including a dementia specific health-related quality of life questionnaire (the DEMQOL) and a generic health-related quality of life questionnaire (the EQ-5D-5L) which capture distinct aspects of health-related quality of life in residential aged care [35]. Upon comparing the included population with the national statistics for people residing in permanent aged care in Australia [25], we found the included population had a similar average age and proportion of females, but there was a slightly higher proportion of people with a diagnosis of dementia in this study.

### Limitations

The facilities which demonstrated a home-like model of care described in this study were all from the same state in Australia, operated by the same provider and are more likely to include people with dementia and potentially more severe behavioural problems. However, in Australia admission to residential aged care facilities is often based on availability at the time of need. Furthermore, 40% of eligible participants declined to participate in the study and therefore the sample may not fully represent the eligible population. We have limited any bias by controlling for confounding factors. Also, all of the recruited organisations were not-for-profit aged care providers.

As a high proportion of people in the study had moderate to severe cognitive impairment proxy measures were used. Although self-assessment is preferable where possible, proxy measures of quality of life are a practical option in residential aged care [35], however, proxy bias may be a factor in this study. The facility-level questionnaire has not been previously validated in an Australian setting and therefore there is further potential for information bias. This is a longer questionnaire and although this was completed by the research assistants hired by the study, not the participants, bias may be introduced as the respondent’s fatigue. Furthermore, the gold standard for measuring medications administered to residents in aged care facilities would be data collected from medication charts, rather than dispensing data; however, it was not feasible to collect this data for all participants in this study. Due to the cross-sectional nature of the data, we cannot assess causality and the direction of association. Therefore, the lower quality of life seen in this study amongst those taking psychotropic medications may be leading to the prescriptions of the medications rather than the medications causing the lower quality of life.

## Conclusions

Older people often report that optimal quality of life is a major priority. Psychotropic medications were associated with lower quality of life in this study. Health care practitioners and consumers should consider this association, and the existing evidence of harm vs benefit of psychotropic medicines, when treating older people living in residential aged care facilities. Adoption of a home-like environment model for residential care may help to reduce the need for psychotropic medications, but this would need to be further explored.

## Additional file

Additional file 1: Table S1. The different psychotropic medications, defined according to the Australian Medicines Handbook, prescribed to the participants of the INSPIRED Study (*n* = 537). (DOCX 15 kb)

## Abbreviations

BPSD: Behavioural and Psychological Symptoms of Dementia
DEMQOL: Dementia Quality of Life questionnaire
EQ-5D-5L: EuroQol-5 Dimensions-5 Levels
INSPIRED: Investigating Services Provided in the Residential Environment for Dementia
NPI: Neuropsychiatric Inventory
PAS-Cog: Psychogeriatric Assessment Scale-Cognitive Impairment Scale
