# Supplementary material for: Psychotropic medications in older people in residential care facilities and associations with quality of life: a cross-sectional study
Source: BMC Geriatr. 2018 Feb 26;18:60. doi: 10.1186/s12877-018-0752-0 (PMC6389148; doi:10.1186/s12877-018-0752-0)
Supplement: Supplementary file 1 — Table S1. The different psychotropic medications, defined according to the Australian Medicines Handbook, prescribed to the participants of the INSPIRED Study (n = 537). (DOCX 15 kb) [file 12877_2018_752_MOESM1_ESM.docx]

Table S1. The different psychotropic medications, defined according to the Australian Medicines Handbook, prescribed to the participants of the INSPIRED Study (n=537).

| **Antipsychotics (24.8%)** | **Antidepressants**  **(49.9%)** | **Benzodiazepines**  **(30.5%)** |
| --- | --- | --- |
|  |  |  |
| Aripiprazole  Chlorpromazine  Flupentixol  Haloperidol  Olanzapine  Periciazine  Quetiapine  Risperidone | SSRIs:  Citalopram  Escitalopram  Fluoxetine  Paroxetine  Sertraline  Tricyclic antidepressants:  Amitriptyline  Doxepin  Serotonin and noradrenaline reuptake inhibitors:  Desvenlafaxine  Duloxetine  Venlafaxine  Other antidepressants:  Mianserin  Mirtazapine | Alprazolam  Bromazepam  Clonazepam  Diazepam  Lorazepam  Midazolam  Oxazepam  Flunitrazepam  Nitrazepam  Temazepam |
